# Mesenchymal stem cell (MSC) homing to kidneys is suppressed by inhibiting interleukin 1-α, tumor necrosis factor-α, or cyclooxygenase-2 signaling

**DOI:** 10.1186/2050-5736-3-S1-O73

**Published:** 2015-06-30

**Authors:** Scott Burks, Ben Nguyen, Saejeong Kim, Michele Bresler, Pamela Tebebi, Joseph Frank

**Affiliations:** 1 NIH Clinical Center, Bethesda, Maryland, United States

## Background/introduction

Maximal homing of iv-infused MSC may be critical for cell therapies. Molecular responses from the primarily mechanical effects of pulsed focused ultrasound (pFUS) (i.e., mechanotransduction) in healthy or diseased murine kidneys generate a “molecular zip-code” consisting of local increases in chemoattractants (cytokines, chemokines, cell adhesion molecules) to enhance MSC homing. These findings have substantial potential to improve cell therapies for regenerative medicine. Since molecular signaling post-pFUS drives enhanced MSC homing, other drugs also aiming to treat disease could potentially interfere with molecular responses and subsequent cell migration to targeted tissue thus undermining cell therapy approaches. This study characterized temporal molecular changes post-pFUS to identify critical signals that drive larger changes observed in the chemoattractants and investigates whether inhibition of the early signals could suppress MSC homing to kidneys.

## Methods

C3H mice received unilateral kidney pFUS (1MHz, 5MPa, 10 ms pulses, 5% duty cycle, VIFU 2000) and kidneys were harvested for ELISA from 10min–72hr after. Pretreatment with drugs included: saline; ibuprofen (nonspecific cyclooxygenase [COX] inhibitor; 30mg/kg, po) 15min pre-pFUS; etanercept (tumor necrosis factor-α inhibitor; 100μg, ip) 72 and 24hr pre-pFUS; or Anakinra (interleukin-1α [IL-1α] inhibitor; 200μg, ip) 48, 24, and 1hr pre-pFUS. Drug-treated kidneys were harvested from 10min–24hr. MSC homing in normal or drug-treated mice included 106 human MSC iv 3hr after kidney pFUS.

Kidneys were harvested 24hr post-injection and MSC were detected by immunofluorescence. Cell counts from pFUS-treated kidneys were compared to untreated contralateral kidneys and ANOVA was used for statistical analysis (p<0.05).

## Results and conclusions

**Figure 1:**
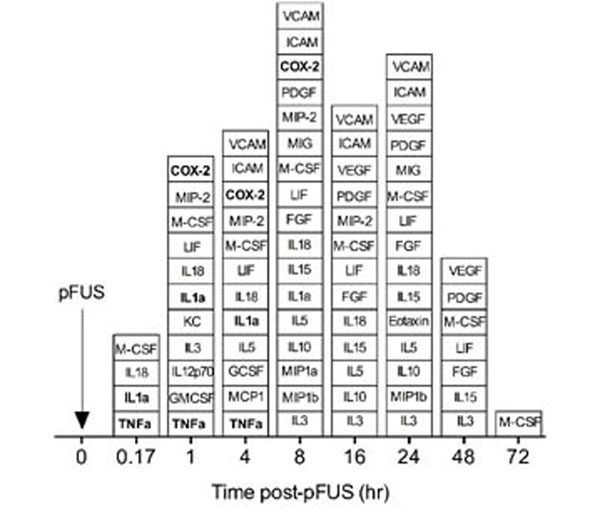
Bloxplot representation of statistically significant (p<0.05 by ANOVA) elevations in cytokines, chemokines, growth factors, and cell adhesion molecules following pFUS to normal kidneys. Responses enhance MSC homing and are inhibited by clinical drugs.

Proteomic analyses of pFUS-treated kidneys revealed early elevations of TNFα and IL-1α (10min), followed by COX2 upregulation shortly thereafter (1–4hr). Thus lead to chemoattractant elevations that induce MSC homing (2–24hr) that returned to baseline by 72hr (Fig 1). Kidneys in mice pretreated with ibuprofen, etanercept, or anakinra, did not generate the molecular zip-code after pFUS. Infused MSC did not home to pFUS-treated kidneys. MSC also failed to home to pFUS-treated kidneys in COX2-knockout mice, demonstrating ibuprofen inhibited COX2 specifically. Drug effects on cell homing remain unexplored and uncontrolled for in cell therapy trials. The molecular zip-code generated by pFUS is driven by early elevations of TNFα and IL1α and COX2 expression. Inhibiting any of these molecules with clinically relevant drugs abrogates the pFUS-induced molecular response and suppresses MSC homing to kidneys. These findings suggest drug-host interactions could undermine cell-based therapies in regenerative medicine. Furthermore, in-depth study of pFUS molecular responses may help elucidate MSC homing mechanisms and pFUS could be a useful platform to screen drug-homing interactions.

